# Hormonal therapy in uterine sarcomas

**DOI:** 10.1002/cam4.2044

**Published:** 2019-03-21

**Authors:** Yuqin Zang, Mengting Dong, Kai Zhang, Chao Gao, Fei Guo, Yingmei Wang, Fengxia Xue

**Affiliations:** ^1^ Department of Gynecology and Obstetrics Tianjin Medical University General Hospital Tianjin China

**Keywords:** aromatase inhibitors, gonadotropin‐releasing hormone analogue, hormonal therapy, progestins, uterine sarcomas

## Abstract

Uterine sarcomas (USs) are a group of rare but aggressive uterine malignancies, accounting for only 1% of the malignant tumors of female reproductive organs. Due to the high rate of recurrence and metastasis, the prognosis of USs is poor. Given the high mortality rate and limited clinical benefit of surgery and adjuvant chemoradiotherapy, hormonal therapy has shown good prospects in recent years. Hormonal agents include progestins, aromatase inhibitors (AIs), and gonadotropin‐releasing hormone analogue (GnRH‐a). According to the literature, hormonal therapy has been confirmed effective for recurrent, metastatic or unresectable low‐grade endometrial stromal sarcoma (LGESS) and hormone receptor positive (ER+/PR+) uterine leiomyosarcoma (uLMS) with favorable tolerance and compliance. Besides, hormonal therapy can also be used in patients with early‐staged disease who desire to preserve fertility. However, due to the rarity of USs, the rationale of hormonal therapy is generally extrapolated from data of hormone‐sensitive breast cancer, and present studies of hormonal therapy in USs were almost limited to case reports and small‐sized retrospective studies. Therefore, further systematic researches and standardized clinical trials are needed to establish the optimal hormonal therapy regimen of USs. Herein, we reviewed the existing studies related to the hormonal therapy in USs in order to provide reference for clinical management in specific settings.

## 1 INTRODUCTION

Uterine sarcomas (USs) are a group of rare mesenchymal malignant tumors, accounting for 3%‐7% of uterine cancers and approximately 1% of female genital tract cancers.^1^, ^2^ The clinical presentation of USs is nonspecific, such as irregular vaginal bleeding, abnormal vaginal discharge, pelvic mass, and abdominal pain. Distant metastasis is prone to developing at the early stage and recurrence is common. And there is a lack of consensus on optimal treatment regimen due to the rarity and histopathological diversity. Taken together, the difficulty of diagnosis, aggressive biology, and no normalized therapeutic maneuver result in the poor prognosis of USs, with the 5‐year survival rate of 31%‐64%.^3^

Based on the updated (4th) edition of the *WHO classification of tumors of female reproductive organs* in 2014, USs contain low‐grade endometrial stromal sarcoma (LGESS), high‐grade endometrial stromal sarcoma (HGESS), uterine leiomyosarcoma (uLMS), undifferentiated uterine sarcoma (UUS), and several rarer types including adenosarcoma, rhabdomyosarcoma (RMS), and perivascular epithelioid cell tumor (PEComa).^2^ A systematic review of studies from 1970 to 2011 demonstrated that uLMS was the most common subtype (63%), followed by ESS (only means LGESS) (21%) and some rarer types such as adenosarcoma (6%), UUS (5%) and other types (5%).^4^ Compared with the third edition, HGESS, an ESS variant with a unique genetic rearrangement YWHAE‐FAM22A/B, was identified as a separate type due to its more aggressive behavior and poorer outcome. Carcinosarcoma, also called malignant mixed mullerian tumor (MMMT), was previously categorized in sarcoma, but is now considered, staged and treated as high‐grade endometrial cancer.^5^

Primary treatment of USs is surgical staging with total hysterectomy (TH) ± bilateral salpingo‐oophorectomy (BSO),^6^ while the lymphadenectomy is controversial. Adjuvant treatments are offered to patients depending on the histopathological type and surgical staging (commonly the 2009 FIGO staging). Adjuvant chemotherapy is beneficial to the control of systemic disease at advanced stages and is recommended to patients with non‐LGESS and uLMS without response to hormone. Postoperative radiotherapy could promote the control of local lesion, but has no appreciable or consistent improvement in the overall survival (OS).^1^ By contrast, hormonal therapy has been found effective to inhibit the growth, decrease recurrence rate and improve survival in a portion of patients with Uss.^7^ Furthermore, compared with other systematic drugs, hormonal agents can be easily administered and possess a tolerable side effect profile, which allows patients to be administered for prolonged periods.

USs exhibit a variable rate of estrogen receptor (ER) and progesterone receptor (PR). A study (n = 291)^8^ in 2016 revealed that ER and PR were expressed in 53% and 67% of LGESS，45% and 65% of uLMS, 23% and 31% of HGESS, and 47% and 63% of all USs, respectively. Objective response and prolonged survival are usually associated with positive expression of hormone receptor. Highest frequency of ER and PR expression was detected in LGESS of all types USs. Understandably, the response of LGESS to hormonal therapy is the best. The expression rate of ER/PR in uLMS is lower than LGESS, but uLMS with receptor expression (ER+/PR+) is sensitive to hormonal therapy. HGESS tends to recur early and frequently (usually <1 year), leading to an extremely poor prognosis.^9^ UUS is another high‐grade sarcoma, with 60% of the patients diagnosed at very late stage (FIGO stage III/IV), and the OS is usually no more than 2 years.^5^ On account of the aggressive nature and rarely expressed ER/PR of HGESS and UUS, hormonal therapy is of little effect to them two. Therefore, the major setting of using hormonal therapy is in patients with LGESS or ER/PR positive uLMS, especially with small volume or indolent growth rate. The commonly used drugs are progesterone, aromatase inhibitors (AIs) and gonadotropin‐releasing hormone analogue (GnRH‐a). Besides, selective estrogen receptor modulators (SERMs) had also been used.

## 2 THE MECHANISM OF HORMONAL THERAPY IN TREATING ESTROGEN‐DEPENDENT UTERINE SARCOMAS

### 2.1 Progestins

Progestins exert an antiestrogenic activity by binding to PR, and eventually cause a decrease in endometrial gland and stromal proliferation.^7^, ^10^ First, progestins induct the 5‐α‐reductase activity in the liver which inhibits the conversion from androgen to estrogen resulting in the reduction of circulating estrogens. Second, progestins increase estrogen metabolism and clearance by upregulating the estradiol 17β‐dehydrogenase. Third, progestins inhibit estrogen‐mediated growth factors, downregulate the ER and thereby inhibit the growth‐stimulating effects of estrogen on ER‐positive cells. Finally, progestins inhibit the production of luteinizing hormone (LH), adrenocorticotropic hormone (ACTH), and other growth factors by a negative feedback action to the adenohypophysis. Moreover, progestins can suppress cyclin‐dependent kinase (CDK) and enhance P27 (CDK inhibitors), then inhibit the binding of CDK to cyclin and block the cell cycle progression, and hence cause the inhibition of cell proliferation.^11^ The commonly used progestins are megestrol acetate (MA) and medroxyprogesterone acetate (MPA).

### 2.2 AIs

Aromatase, encoded by the gene of CYP19, is a member of cytochrome P450 superfamily. It is the key enzyme of estrogen biosynthesis, which catalyzes the conversion of androstenedione and testosterone to estrone and estradiol.^12^ Aromatase is broadly expressed in ovary, placenta, breast, skin, adipose, and other tissues.^12^ For postmenopausal women, adipose tissue is the main source of aromatase. It was reported that intra‐tumoral aromatase was expressed in approximately 80% of LGESS^13^ and 60% of uLMS.^7^ AIs not only reduce the level of estrogen in the circulation by inhibiting the activity of aromatase in peripheral tissues, but also inhibit the biosynthesis process of estrogen within tumor tissues.^14^ Based on the molecular structures and mode of action, AIs are divided into two types: steroidal AIs and non‐steroidal AIs. The former one, which is also called “irreversibly inhibitors” or “suicide inhibitors”, possesses similar structures to androgens and can irreversibly bind to aromatase. While the latter one which inhibits the activity by reversibly binding to the heme group of aromatase, are known as “reversibly inhibitors” or “competitive inhibitors.”^14^, ^15^ On the grounds of time in use, AIs contain three generations.^10^, ^16^, ^17^ First‐generation (aminoglutethimide) and second‐generation (formestan and fadrozole) AIs are nonspecific non‑steroidal AIs with serious side effects on account of the inhibition of mineralocorticoid and glucocorticoid synthesis. The third‐generation AIs including non‐steroidal letrozole and anastrozole and steroidal exemestane, have minimal effects on the adrenal glands and can be orally administered.

### 2.3 GnRH‐a

GnRH‐a is a class of synthetic ramification of GnRH with a similar structure to GnRH but more stability, longer half‐life, and greater affinity to GnRH receptor. GnRH‐a competes the binding site of GnRH receptor with GnRH and inhibits the secretion of Gn from hypophysis leading to the reduction of level of estrogen. When GnRH or GnRH‐a is increased, follicle‐stimulating hormone (FSH) and LH would also increase in a short time. Then, the level of GnRH receptor would reduce, causing a profound decrease of sensitivity of hypophysis to GnRH. In consequence, the secretion of FSH and LH is decreasing leading to the lower level of estrogen.^18^ It has been demonstrated that intra‐tumoral GnRH receptor was expressed in about 80% of ESS, which indicated that GnRH‐a may be put into action by blocking these GnRH‐R.^19^ What's more, studies both in vivo and vitro^20^, ^21^ demonstrated that GnRH‐a could not only inhibit the proliferation of ovarian cancer cells by improving the level of inositol phosphate and activating protein kinase pathways such as ERK1/2, but also induce the apoptosis of ovarian cancer cells by upregulating the apoptosis associated gene and activating Fas system.

### 2.4 SERMs

SERMs are a series of compounds that act on the estrogen receptor, mainly including non‐steroidal tamoxifen and toremifene and steroidal fulvestrant. Tamoxifen, acting as the ER antagonist in breast tissue leading to the inhibition of the activity of estrogen, is widely used in treating breast cancer. However, it has been recognized that tamoxifen and toremifene might exert an estrogen‐like effect in uterus which promote the development of endometrial cancer. The opposite effect of SERMs in breast and uterus may due to the different expression of co‐regulatory proteins. In breast, tamoxifen could recruit the co‐inhibitors at the target promoter site and play an anti‐estrogenic role; while in uterus, it could recruit the co‐promotors and play an estrogenic role leading to the promotion of endometrial tumors.^22^, ^23^

## 3 HORMONAL THERAPY IN LGESS

### 3.1 Overview of LGESS

LGESS is an inert tumor with the 5‐year OS up to 71.8%.^24^ Yet, patients with LGESS usually recur in 10‐20 years after initial diagnosis (36%‐56%),^25^ and 15%‐25% of patients die from the recurrence.^6^ TH±BSO is the mainstay of treatment. But for young nulliparous women at early stage, preservation of fertility can be considered. Although BSO is favored for LGESS, ovaries can be preserved for premenopausal patients with stage I LGESS, because it does not compromise survival.^2^, ^26^ In view of the little impact on survival, systematic lymphadenectomy may not be recommended in patients with LGESS unless the patient has obvious evidence of extrauterine involvement, clinically suspicious enlarged nodes, or advanced disease.^27^ Chemotherapy and radiotherapy are important adjuvant treatment for advanced LGESS, but no potent evidence of benefit to prognosis is established. Whereas, hormonal therapy shows an expectant effect on treating LGESS no matter in the early or advanced stage. Previous studies reported a mortality of 19%‐50% while recent studies suggested a mortality of <10%, and meaningfully, this decrease is mainly attributed to the application of hormonal therapy.^28^

### 3.2 Progestins in LGESS

With a relative high response rate and prolonged time to progression, progestins are the most frequently used first‐line hormonal therapy in LGESS. According to the literature, progestins were used in LGESS in the following settings: (a) postoperative adjuvant treatment; (b) treatment for recurrent and metastatic disease; (c) fertility‐sparing treatment.

For example, Beck et al^29^ found that the recurrence rate of patients with postoperative progestogen was lower than those with surgery alone in stage I (14.3% vs 38.5%), as well as all stages (33% vs 50%). In total, seven retrospective studies were searched in Pubmed in this respect, and six of them came to the conclusion that patients who had progestogen as adjuvant treatment had a lower recurrence rate than those who had surgery alone. The patients kept no evidence of disease for 18‐56 months.

As for progestins treatment for recurrent or metastatic LGESS, there were several case reports and retrospective studies on Pubmed. As shown in Table 1, the total clinically effective rate was 86.9%. The longest duration of response was 252 months. Due to the known bias of case reports (that is, published case reports were usually with positive outcomes, which would raise the effective rate), we did not include them in the table. Thus, progestins can indeed inhibit disease progression effectively and prolong the survival of recurrent or metastatic patients.

**Table 1:** Case series and retrospective studies of progestins in the setting of recurrent, metastatic, or unresectable LGESS

Study (y)	n	Age	Hormonal treatment	Response	Duration (mo)
Chu (2003)^56^	8	–	progestins	4 CR, 3 SD, 1 PD	18‐180
Pink (2006)^22^	3	42‐63	MPA	1 CR, 1 SD, 1 PD	0‐50
Dahhan (2009)^57^	8	27‐46	MA	4 CR, 3 PR, 1 SD	18‐252
Ioffe (2009)^49^	5	–	MA, MPA	1 PR, 3 SD, 1 PD	6‐124
Mizuno (2012)^58^	6	32‐53	MPA	3 PR, 3 SD	26‐146
Yamazaki (2015)^28^	8	50‐69	MPA	3 CR, 2 PR, 1 SD, 2 PD	–
Total	38		progestins	12 CR (31.6%), 9 PR (23.7%), 12 SD (31.6%), 5 PD (13.2%) Effective rate: 86.9%	

CR, complete response; MA, megestrol acetate; MPA, medroxyprogesterone acetate; PD, progression of disease; PR, partial response; SD, stable disease; —, the data were not described clearly.

Effective rate: CR+PR+SD.

Fertility‐sparing management has been gradually common in young nulliparous women with stage I LGESS (ER+/PR+) recently, and progestins are the primary drugs. Relevant studies are listed in Table 2. From the table, we can learn that all patients with fertility desire in these studies were successfully conceived except for one study^30^ in which the patients’ fertility desire were not sure. And only 1 of the 15 pregnancies was ended in abortion. Assisted reproductive technology (ART) were performed in 3 of the 15 pregnancies (20%). Visibly, the effect of progestins in fertility‐sparing treatment is favorable. Nevertheless, considering the tendency of late recurrence of LGESS, regular follow‐up should be performed during treatment, and prophylactic hysterectomy is recommended after parturition.

**Table 2:** Studies of progestins in the setting of fertility‐sparing treatment of early‐staged LGESS

Study (y)	n	Stage	ER/PR	Hormonal treatment	Treatment duration	Pregnancy desire	Pregnancy	Successful parturition	Management after pregnancy and outcome
Stadsvold (2005)^59^	1	IB	1 +/+	MA 100 mg/d	–	0	0	0	No TH±BSO, NED 21 mo
Sanchez (2012)^60^	1	IB	1 +/+	MA 160 mg/d	–	2	2 (1 by ART)	2	Recurrence after 2 y, TH+GnRHa+MA 160 mg/d, NED 5 y
Delaney (2012)^61^	1	IB	1 +/+	MA	8 y	1	1	1	No TH±BSO, NED 8 y
Zhan (2014)^62^	1	IB	–	CT+MPA 250 mg/d	7 mo	1	1	1	No TH±BSO, NED 47 mo
Morimoto (2015)^63^	1	–	1 +/+	MPA 600 mg/d	39 mo	0	0	0	Recurrence after 39 mo, TH+BSO+RT+CT+GnRHa, DOD
Dong (2014)^64^	1	–	1 +/+	MPA 250 mg/d	1 y	1	1	1	No TH±BSO, NED about 3 y
Laurelli (2015)^30^	5	5 IA	5 +/+	MA 160 mg/d	1‐2 y	–	2	1	1 No TH±BSO, 4 TH+BSO, NED 30‐54 mo
Jin (2015)^65^	4	–	4 +/+	MA 160‐320 mg/d	5‐6 mo	3	3 (1 by ART )	3	3 No TH±BSO, NED 13‐36 mo 1 Recurrence after 10 mo, TH+MA 320 mg/d, NED 10 mo
Xie (2016)^26^	10	3 IA 7 IB	–	8 MA/MPA 2 CRS+MA+CT^a^	–	5	5 (1 by ART )	5	5 No TH±BSO, NED 4‐106 mo 1 Recurrence after 4 mo, FSS+MA, NED 39 mo 2 Recurrence after 3‐18 mo, TH+BSO+GnRHa, NED 13‐32 mo 1 Recurrence after 26 mo CRS+MA+CT, NED 77 mo 1 Recurrence after 24 mo, local tumorectomy, NED 48 mo
Total	25			MA/MPA			15	14	NED 4‐106 mo

ART, assisted reproduction technology; BSO, bilateral salpingo‐oophorectomy; CRS, cytoreductive surgery; CT, Chemotherapy; DOD, death of disease; FSS, fertility‐sparing surgery; MA, megestrol acetate; MPA, medroxyprogesterone acetate; NED, No evidence of disease; RT, Radiotherapy; TH, total hysterectomy; —, the data were not described clearly.

a After the primary fertility‐sparing treatment, recurrence was found during pregnancy in two patients who underwent cesarean delivery and cytoreductive surgery followed by adjuvant MA and chemotherapy.

### 3.3 AIs in LGESS

AIs used to be the second‐line hormonal therapy after the failure of progestins treatment in recurrent LGESS. The first study on this aspect was reported in 2001. But recently, owing to the superiority of efficacy and more acceptable safety profile over progestins, the third‐generation AIs have also been used as first‐line hormonal treatment. Searching on the PubMed, six case series and retrospective studies about the use of AIs as second‐line therapy for recurrent, metastatic, unresectable, or progestin‐resistant LGESS were obtained (Table 3). The case reports were excluded for the bias. In total, the outcomes after treatment were described in 29 patients, with a clinical effective rate of 89.7%. Additionally, excluding the case reports, there were five studies reported the outcome of AIs as first‐line therapy, with a clinical effective rate of 91.7% (Table 3). To conclude, AIs show nearly equivalent or even better response as the first‐line therapy than as second‐line therapy. And the longest duration of response is 168 and 124 months, respectively.

**Table 3:** Case series and retrospective studies of AIs in the setting of recurrent, metastatic, or unresectable LGESS

Study (y)	n	Age	ER/PR	Hormonal treatment	Response	Duration (mo)
Pink (2006)^22^	3	42‐69	3 +/+	3 Letrozole (2.5 mg/d)	3 PR	–
Ioffe (2009)^49^	2	–	–	2 Letrozole	1 PR, 1 SD	53
Dahhan (2009)^57^	1	53	–	1 Letrozole (2.5 mg/d)	PR	>4
Yamaguchi (2015)^17^	5	36‐70	5 +/?	5 Letrozole (2.5 mg/d)	2 CR, 1 PR, 2 SD	–
Ryu (2015)^10^	13	42‐87	10 +/+	12 Letrozole (2.5 mg/d) 1 Aminoglutethimide (500 mg qid)	5 CR, 6 PR, 2 PD	4‐168
First‐line therapy	24			AIs	7 CR, 12 PR, 3 SD, 2 PD Effective rate: 91.7%	
Spano (2003)^23^	2	43‐53	2 +/+	1 Triptorelin → Aminoglutethimide (500 mg qid) 1 Aminoglutethimide +cortisol → Letrozole (2.5 mg/d)	2CR	84‐168
Pink (2006)^22^	2	59‐69	2 +/+	2 Letrozole (2.5 mg/d)	1 PR, 1 PD	–
Ioffe (2009)^49^	1	–	1 +/+	1 Letrozole	1 PR	124
Dahhan (2009)^57^	2	47‐87	–	2 Letrozole (2.5 mg/d)	1 PR, 1 PD	–
Altman (2012)^13^	4	28‐44	–	4 Anastrozole (1 mg/d) 1 Anastrozole (1 mg/d) → Letrozole (2.5 mg/d) 1 Anastrozole (1 mg/d) → Exemestane 25 mg/d	3 SD, 1 PR	–
Ryu (2015)^10^	18	28‐63	10 +/+	9 Letrozole (2.5 mg/d) 7 Anastrozole (1 mg/d) 1 Aminoglutethimide (500 mg qid) 1 Exemestane 25 mg/d	3 CR, 10 PR, 4 SD, 1PD	3‐124
Second‐line therapy	29			AIs	7 CR, 13 PR, 6 SD, 3 PD Effective rate: 89.7%	
Total	53			AIs	14 CR, 25 PR, 9 SD, 5 PD Effective rate: 90.6%	

CR, complete response; PD, progression of disease; PR, partial response; SD, stable disease; —, The data were not described clearly; →, It means a change from the former one to the latter one.

Effective rate: CR+PR+SD.

Similar with progestins, AIs can also be applied to fertility‐sparing treatment in early LGESS patients. But there is only one study reported by Choi MC et al.^31^ A 31‐year‐old nulliparous LGESS women with strongly expressed ER and PR, received adjuvant therapy with letrozole for 6 months after fertility‐sparing surgery. Finally, after 32 months conservative treatment, she conceived by in vitro fertilization and delivered twins at 32^+2^ weeks gestation by cesarean section. No evidence of recurrence was found during the 99 months of follow‐up.

### 3.4 GnRH‐a in LGESS

Mesia et al^32^ reported one patient who was preoperatively considered to be uLMS. After treated with leuprolide acetate (3.75 mg/month) for 2 months, her tumor was reduced in size, and postoperative pathology was confirmed as LGESS combined with uLMS. Alkasi et al^33^ reported a case of LGESS patients with multiple metastatic lesions in the lung during the 8th and 9th year after operation. She then underwent lung resection surgery, and GnRH‐a was given to her for 2 years. Complete relieve was achieved eventually. In a more recent study,^34^ a stage IB LGESS patient was treated with TH+BSO and leuprolide acetate (3.75 mg/28 day) for 6 months. Finally, the patient achieved complete relieve. In the study of Xie W et al,^26^ GnRH‐a was used in 4 stage I cases as adjuvant therapy after fertility‐sparing surgery, of which 3 cases had fertility intention but failed to pregnant. Another 2 patients with stage I disease relapsed after conservative treatment and GnRH‐a was administrated after TH+BSO, ending in no recurrence. Thus, used as a single agent in LGESS, GnRH‐a achieves a reduction in tumor volume before surgery, and prevents recurrence and metastasis as well as prolongs survival after surgery. However, the effect of postoperative fertility preservation was not satisfactory.

In addition to the monotherapy of GnRH‐a, the combination with other hormonal drugs was also common in LGESS. GnRH‐a and progestins or AIs was the frequently used combination. Dupont et al^35^ reported one case of stage IA patients who developed metastases in the lungs 1 year after TH+BSO. She was treated with MA (80 mg, qd) combined with GnRH‐a (7.5 mg/28 day) and remained no evidence of recurrence for 8 years. In the study of Xie et al,^26^ two stage IA patients were treated with GnRH‐a and Mirena as postoperative adjuvant therapy, and remained disease‐free for 8 and 33 months, respectively. In another study,^17^ a 36‐year‐old patient with lung metastasis achieved partial relieve after treatment with letrozole and GnRH‐a.

### 3.5 SERM in LGESS

Studies have shown that the use of tamoxifen postoperatively can lead to disease progression in ESS patients, as well as the development of ESS in breast cancer patients.^22^, ^23^, ^36^ In 2014, the European Society for Medical Oncology (ESMO) guidelines noted that tamoxifen and HRT is contraindicated drugs of ESS treatment.^37^ Yet, fulvestrant, the pure estrogen alpha receptor inhibitor, has been reported successfully reducing the tumor diameter in an LGESS (ER+) patient who refused surgery but developed multiple metastases after treated with progesterone and anastrozole.^38^

## 4 HORMONAL THERAPY IN ULMS

### 4.1 Overview of uLMS

Due to the mimic appearance of uterine leiomyoma (uLMY) and low diagnostic accuracy of endometrial sampling, it is challenging to diagnose preoperatively for uLMS.^39^ A large population‐based study declared that the 5‐year survival of uLMS was 41.9%,^24^ while in advanced uLMS it was less than 15%.^40^ For early uLMS, TH±BSO is the fundamental treatment. With no increase of mortality, ovaries can be preserved for premenopausal women with a sarcoma limited to the uterus.^41^ As with LGESS, lymphadenectomy is also not recommended for early‐staged uLMS. As reported, 50%‐71% of patients suffer a relapse after surgical resection.^42^ Adjuvant chemotherapy and radiotherapy both confer a little survival advantage.^39^, ^40^, ^43^ Targeted drugs such as pazopanib, trabectedin, and eribulin are usually used when traditional therapies were failed.^44^ With a high expression rate of ER and PR, uLMS shows good response to hormonal therapy.^40^ Version 2. 2015*, NCCN Clinical Practice Guidelines in Oncology* recommended AIs to ER/PR positive uLMS for the first time; and the guideline of Version 1. 2016 added other hormone medicines including MA, MPA, and GnRH‐a to the hormonal therapy of uLMS.

### 4.2 Progestins in uLMS

Based upon the study of Uchida et al,^45^ a 51‐year‐old woman with a past history of uLMY was diagnosed as metastatic uLMS (ER+/PR+) in lung. One month postoperatively, she received MPA, 600 mg daily, for one course. Afterwards, the residual lesion in her chest diminished gradually, and she has remained well on this MPA regimen for 45 months. Similarly, Lo et al^46^ has reported a case of a 58‐year‐old patient with pelvic uLMS mess (ER+/PR+) and pulmonary nodules who had suffered the TH+BSO for uLMY 6 years ago. After the surgical resection, the treatment of MPA 200 mg daily was commenced, she then maintained tumor free for 12 months. However, both the two reports were published more than 10 years ago, and there has been no relevant report in recent years. It was declared that progestin can either promote or inhibit growth of uLMY in vitro, depending on the culture conditions.^47^ On the one hand, in animal studies, the uLMY induced by exposure to exogenous estrogen can be inhibited by progesterone. On the other hand, higher doses (5 mg/day) of MPA were spotted to increase the growth of uLMY significantly. Furthermore, progestins could attenuate or reverse the inhibitory effects of GnRH‐a on leiomyoma size. Therefore, the role of progesterone in uLMS is still ambiguous, and the use of it to uLMS should be cautious.

### 4.3 AIs in uLMS

The first report about AIs using in uLMS was in 2007. In recent years, there has been more reports about the application of AIs in uLMS, mainly including two aspects: (a) postoperative adjuvant therapy for patients with stage I uLMS; (b) treatment for patients with recurrent, metastatic, and unresectable uLMS. Letrozole is the uppermost first‐line hormone drug, while exemestane and anastrozole are normally used as the second‐line therapy. Different from LGESS, there has been no report of fertility‐preserving treatment in patients with uLMS at present.

Stage I uLMS is limited to the uterus, but the recurrence rate is still high (>50%).^48^ Thus, the postoperative adjuvant treatment is really necessary. Reported by Ioffe et al,^49^ 3 patients with ER and PR positive uLMS who received anastrozole or letrozole therapy after surgery, maintained no evidence of disease for 72, 25, and 18 months, respectively. This study indicated that postoperative adjuvant treatment with AIs for patients with early uLMS could prolong the disease‐free time.

Studies also demonstrated that AIs showed an approving effect in the setting of recurrent, metastatic, and unresectable uLMS, especially ER/PR positive ones. All relevant case series and retrospective and prospective studies searched for in Pubmed are listed in Table 4. As is shown, the total clinical efficacy rate is 51%, with the clinical efficacy rate of 70% and 44%, respectively, when used as the first‐line and second‐line therapy. Among these studies, the phase 2 trial of letrozole using in uLMS patients in 2014 by George et al^43^ is the only one clinic trial. All 27 involved patients were postmenopausal women with ER+and/or PR+advanced (metastatic and/or unresectable) uLMS, who have never received prior hormonal therapy for the treatment of uLMS. SD was observed in 54% of all patients and the 12‐week PFS rate was 50%. It is notable that longer PFS was more likely to be observed in patients with strongly expresses ER and PR and this phenomenon was similar in almost all the studies.

**Table 4:** Case series and retrospective and prospective studies of AIs in the setting of recurrent, metastatic or unresectable uLMS

Study (y)	n	Age	ER/PR	Treatment for recurrence or metastasis	Response	Duration (mo)	Prognosis
Ioffe (2009)^49^	4	–	1 +/+ 3 ±	1 Anastrozole 3 Letrozole	3 SD,1 PR	30‐50	–
Thanopoulou (2014)^40^	16	39‐72	16 +/+	13 Letrozole (2.5 mg/d) 2 Letrozole (2.5 mg/d) + Goserelin 1 Letrozole(2.5 mg/d) → Anastrozole(1 mg/d)	2 PR, 8 SD, 6 PD	13	Median PFS: 14 mo
First‐line therapy	20		17 +/+		3 PR, 11 SD, 6 PD Effective rate: 70%		
Altman (2012)^13^	3	44‐48	1 +/+ 2 ±	2 Anastrozole (1 mg/d) 1 Anastrozole (1 mg/d) → Letrozole (2.5 mg/d)	2 SD, 1 PD	4.2 (mean)	Mean OS: 44.3 mo
Thanopoulou (2014)^40^	6	40‐74	6 +/+	5 Exemestane (25 mg/d) 1 Anastrozole (1 mg/d)	3 SD, 3 PD	3	1‐y PFS rate: 80%
George (2014)^43^	27	44‐74	22 +/+, 4 ±	Letrozole (2.5 mg/d)	14 SD, 13 PD	0.4‐9.9	12‐week PFS rate: 50%
Second‐line therapy	36		29 +/+		19 SD, 17 PD Effective rate: 52.7%		
O'Cearbhaill (2010)^a^ ^66^	34	35‐74	23 +/+, 5 ‐/‐, 3 ‐/?	Letrozole Exemestane Anastrozole	3PR, 11SD, 20PD	1‐84	Median PFS: 2.9 mo
Total	90		69 +/+		6 PR, 41 SD, 43 PD Effective rate: 52.2%		

Effective rate: CR+PR+SD.

CR, complete response; OS, overall survival; PD, progression of disease; PFS, progression free survival; PR, partial response; SD, stable disease; —, The data were not described clearly; →, It means a change from the former one to the latter one.

a This study did not mention whether AIs were used as first‐line therapy or as second‐line therapy.

### 4.4 GnRH‐a in uLMS

GnRH‐a was reported to reduce the volume of uLMY by suppressing the synthesis of special collagen.^50^ What's more, binding sites for GnRH‐a have also been described in uLMY.^51^ The first report of the use of GnRH‐a in uLMS was in 1990. Studies^52^, ^53^ indicated that GnRH‐a could relieve the symptoms of patients with uterine smooth muscle tumors but would add complexity to the differentiation from uLMS to benign uLMY, thus delay the diagnosis and treatment and deteriorate the prognosis. Thus, the use of GnRH‐a in uterine smooth muscle tumors should be taken carefully after detailed discussion about the individuals and balancing the benefits of symptom‐control and the risk of delay of the occult uLMS.

### 4.5 SERM in uLMS

The role of SERM in uLMS is similar to that in LGESS. Reported by Ioffe et al,^49^ a premenopausal woman with stage II uLMS received tamoxifen as salvage therapy and remained stable disease for 12 months. Nevertheless, others studies suggested that tamoxifen could promote the genesis and progression of USs. As an example, Samuji et al^36^ declared that 3 patients with breast cancer developed HGESS, carcinosarcoma and uLMS in no long time after or during the hormonal therapy tamoxifen. Reportedly,the incidence of uterine sarcomas would increase to 17/100,000 woman per year in those patients using tamoxifen for >5 years.^7^, ^39^ As a consequence, tamoxifen is contraindicated in uLMS.

## 5 HORMONAL THERAPY IN OTHER TYPES OF UTERINE SARCOMAS

Other types of USs are more rare and less sensitive to hormonal therapy. But there are also several case reports about the application of hormone therapy in these types. In the settings of adjuvant, recurrent or metastatic treatment of uterine adenosarcoma, progestins, AIs, GnRH‐a, and SERMs have all been used. In the light of the reports, response ranged from 10 months to 7 years and was related to the status of ER/PR In 2017, Baek et al 54 reported a patient who was diagnosed as stage III undifferentiated endometrial sarcoma with CYP19A1 expression. She was given letrozole for adjuvant treatment after the surgery and chemotherapy and remained no evidence of disease to the end of study. Okamotoa et al^55^ reported a woman who underwent a video‐assisted thoracoscopic surgery to remove the pulmonary metastases (ER+and PR+) after the surgery of uterine PEComa 3 years ago. She then continued the GnRH‐a therapy and remained no new metastasis or enlargement of the existing lesions for 2 years. For RMS, FSH, and LH receptors were reported to be expressed in its established cell lines and primary tumor tissues isolated from patients.^54^ And the stimulation of pituitary and gonadal sex hormone triggered the enhancement of proliferation, chemotaxis, cell adhesion as well as the phosphorylation of MAPK and AKT signaling in human RMS cell lines. So, GnRH‐a may be prospective to be used in RMS.

## 6 LIMITATIONS AND CHALLENGES OF USING AIS IN UTERINE SARCOMAS

As has been noted above, hormonal therapy has shown prospects in the treatment of USs. Nevertheless, there exists limitations. For one thing, hormonal therapy has an acceptable toxicity profile but the adverse reactions can also not be neglected. For another, due to the fact that USs are rare, evidence for the effect of hormonal therapy are limited and the optimal regimen remains unsure.

Long‐term high‐dose progestin therapy could cause gastrointestinal reaction, severe depression, weight gain, and thromboembolism complications. First and second generation of AIs interfere the production of adrenocortical hormone by inhibiting other CYP450 enzymes in the chain of steroid synthesis, resulting in somnolence, rash, nausea, fever, and other side effects.^16^ The third generation of AIs with high selectivity show little effect on the adrenal glands,^16^, ^17^ but patients using them would suffer hypoestrogenic symptoms such as hot flash, fatigue, arthralgia, and osteoporosis.^10^, ^43^ Long‐term use of GnRH‐a could also raise the hypoestrogenic symptoms.^18^

The rarity of USs multiplies the difficulty of study about hormonal therapy. The exiting studies were all case reports and small‐sized retrospective researches with less convincingness except one phase 2 clinic trial. The dosage of hormonal agents in USs was extrapolated from the data of breast cancer. MA is recommended to be started at 40 mg orally daily and increased gradually to the recommended total dose of 160 mg or, if needed, to 320 mg daily. The recommended dose of MPA was 200 mg daily.^7^ The usual dosages of AIs are 2.5 mg/day of letrozole, 1 mg/day of anastrozole and 25 mg/day of exemestane. GnRH‐a is usually administrated at a dose of 3.75 mg per 4 weeks. Additionally, the duration of hormonal manipulation is also indeterminate. To date, there has been no specific trial of USs about the effectiveness of different agent, dosage or duration. Similarly, the therapeutic effect of the combination of different hormonal drugs is still unclear. In view of the above, more retrospective researches with large size of samples and randomized controlled clinical trials are needed to figure out these issues.

What's more, the combination of hormonal therapy with other adjuvant therapy, especially targeted therapy, is also promising in the treatment of USs. In breast cancer, it was reported that when combined AIs with novel targeted drugs such as mTOR inhibitors, PI3K inhibitors, and CDK4/6 inhibitors, the PFS of patients was significantly increased.^15^ However, there is no similar study in USs. Therefore, in the future, attempt should be made to study the combination of hormonal therapy with other adjuvant therapy in USs.

## 7 CONLUSION

In summary, hormonal therapy shows high therapeutic index with good tolerance and compliance in LGESS and ER/PR positive uLMS. Progestins and AIs are widely used in LGESS in the setting of postoperative adjuvant therapy, fertility‐sparing therapy, and advanced palliative therapy. AIs also exert an effect of relieving symptoms, reducing the risk of recurrence and controlling the progression in uLMS. But the role of progestins in uLMS is controversial. GnRH‐a is usually used in a combination with progestins or AIs and present a good effect in LGESS, but it would delay the diagnosis and treatment in uLMS. Tamoxifen, the representative of SERM, is no longer recommended to USs. However, due to the rarity of USs, studies are almost limited to case reports and retrospective researches with small samples. Therefore, further studies are needed for the evaluation of curative effect of different drugs, dosages, and durations of hormonal therapy as well as the combination with chemotherapy or targeted therapy.

## CONFLICTS OF INTEREST

All authors approved the manuscript and have agreed to submit it to your esteemed journal. There is no conflict of interest to declare.
